# The Terneuzen Birth Cohort. Longer exclusive breastfeeding duration is associated with leaner body mass and a healthier diet in young adulthood

**DOI:** 10.1186/1471-2431-11-33

**Published:** 2011-05-10

**Authors:** Marlou LA De Kroon, Carry M Renders, Michelle PJ Buskermolen, Jacobus P Van Wouwe, Stef van Buuren, Remy A Hirasing

**Affiliations:** 1 Department of Public and Occupational Health, The EMGO Institute for Health and Care Research, VU University Medical Centre, Amsterdam, The Netherlands; 2 Department of Health Sciences, Faculty of Earth and Life Sciences and EMGO Institute for Health and Care Research, VU University, Amsterdam, The Netherlands; 3 Netherlands Organization for Applied Scientific Research, TNO Quality of Life, Prevention and Health Care, Leiden, The Netherlands; 4 Dept of Methodology and Statistics, FSS, University of Utrecht, The Netherlands

## Abstract

**Background:**

Breastfeeding (BF) is protective against overweight and is associated with dietary behaviour. The aims of our study were to assess the relationship between exclusive BF duration and BMI, waist circumference (WC) and waist-hip ratio (WHR) at adulthood, and to study whether dietary behaviour could explain the relationship between BF duration and the proxies of fat mass.

**Methods:**

In 2004-2005, 822 subjects from the Terneuzen Birth Cohort (n = 2,604), aged 18-28 years, filled in postal questionnaires including sociodemographic factors and aspects of dietary behaviour (dietary pattern, and consumption of fruit and vegetables, snacks, sweetened beverages and alcohol); 737 subjects also underwent anthropometric measurements of weight, height, and waist and hip circumference. The relationship between exclusive BF duration and dietary outcomes was investigated by logistic regression analysis. The relationships of BF duration with the anthropometric measures were investigated by linear regression analyses. All results were corrected for age, gender and possible confounders. Finally, regression analyses were performed to investigate if diet factors had a mediating effect on the relationship between BF duration and fat mass.

**Results:**

A significant inverse dose-response relationship of BF duration was found for BMI (β-0.13, SE 0.06), WC (β-0.39, SE 0.18) and WHR (β-0.003, SE 0.001), after correction for age, gender and confounders. The odds ratio (OR) of exclusive BF duration in months for a breakfast frequency of at least 5 times a week was 1.16 (95%CI 1.06-1.27), and for snack consumption of less than twice a week was 1.15 (95%CI 1.06-1.25). Both ORs were corrected for age, gender and confounders. For other dietary outcomes, the results point in the same direction, i.e. a positive relationship with BF duration, but these were not statistically significant. A mediating effect of the diet factors on the association between BF and anthropometric outcomes was not shown.

**Conclusions:**

Exclusive BF duration had a significant inverse dose-response relationship with BMI, WC and WHR at young adulthood. BF duration was positively related to a healthier diet at adulthood, but this did not explain the protective effect of BF against body fat. Our results underline the recommendation of the WHO to exclusively breastfeed for 6 months or longer.

## Background

Children with overweight or a relatively rapid weight gain during growth tend to become overweight adults [1,2] with an associated high risk of cardiovascular diseases, type 2 diabetes and cancer [3,4]. Exclusive breastfeeding (BF) for at least 4 months not only protects the infant against adverse health outcomes, such as eczema [6] and asthma [7], but also against overweight and obesity [8,9]. Several studies have also shown a dose-response relationship between BF and overweight at later ages [9-12], of which two studies assessed this relationship up until adulthood [10-12]. In these studies overweight was defined on the basis of body mass index (BMI) or weight by height. It is not clear how BF is related to waist circumference (WC) and waist-hip ratio (WHR). These are both proxies of visceral fat, which is considered to be extremely harmful to health [12].

Dietary behaviour, which is often developed at a young age, is an important predictor of overweight [13]. Therefore, generally more and more attention is being paid to promoting the development of healthy dietary habits from birth onwards. Breast milk comprises flavors that reflect foods consumed by the mother, and therefore breastfed infants are more accepting than formula-fed infants of new solid foods when they are offered in a later phase of life [14]. BF [15,16] and parental consumption of fruit and vegetables [15,17,18] are both correlated to children's consumption of fruit and vegetables. Two studies have showed that BF is associated with a variety of diet factors at the ages of 12 months and 7 years [16,19]. At both ages breastfed children consumed fruit and vegetables more frequently than non-breastfed children. These studies did not assess the relationship of BF with other dietary habits such as eating breakfast or having a regular eating pattern. Moreover, it is not known whether the relationship between BF and consumption of healthy food products still exists at young adulthood, or whether this relationship partly explains the relationship between BF duration and accumulation of fat mass.

The aims of our study were 1.) to assess the relationship between exclusive BF duration and anthropometric measures approximating body fat or visceral fat, i.e. BMI, WC and WHR, and dietary behaviour at young adulthood, and 2.) to determine to what extent dietary behaviour explains the relationship of BF duration with body and/or visceral fat mass.

## Methods

### Study population and study design

We analyzed data from the Terneuzen Birth Cohort (n = 2,604). A more detailed description of the study population and study design has been given previously [2,20]. In 2004-2005, 822 subjects with an age range of 18-28 years (31.6%) participated in a follow-up study at young adulthood: they completed questionnaires and 762 subjects of them also participated in anthropometric measurements. The questionnaires included data on sociodemographic characteristics and dietary behaviour. These adults' mothers also completed a questionnaire. After excluding children with missing data for BF (n = 12), the data for 810 young adults were used for statistical analyses. These subjects were representative of the original cohort regarding birth weight and parity of the mother (p > 0.05). However, significant differences with the original cohort existed for gender (42% males vs. 55% in the original cohort), the duration of exclusive BF (mean duration 53 days vs. 45 days in the original cohort), and the age of the mothers (mean 26.9 vs. 26.6 years in the original cohort). The study protocol was approved by the Medical Ethics Committee of the VU University Medical Centre Amsterdam, and written informed consent was obtained from all the participants.

### Physical examinations

Physical examinations were performed by two assistants who received standardized training at the Municipal Health Services in Terneuzen, the Netherlands. Weight was measured, with the subject in underwear, to the nearest 0.1 kg on an electronic self-zeroing scale. Standing height was measured to the nearest 0.1 cm with the aid of a stadiometer. WC was measured mid-way between the lower side of the lowest rib and the upper side of the pelvis, on bare skin, after a normal expiration, and with muscles relaxed.

### Independent variables: BF duration and confounders

Data about BF were prospectively collected from birth until the age of 6 months during the visits by the mothers and their babies to the Child Health Care clinics of the Municipal Health Services in Terneuzen. These visits took place at 1, 2, 4, 8, 10, 14 weeks and 5 and 6 months of age. At each visit it was recorded if the baby received exclusive BF, a combination of BF and formula milk, or only formula milk. Also the date of the introduction of formula milk and the last day of BF were recorded. The duration of BF was defined as the duration of exclusive BF, and was categorized as 0-15, 16-45, 46-74, 75-104, 105-134. 135-164 and ≥ 165 days. This corresponds to 0, 1, 2, 3, 4, 5 and 6 months respectively (rounded to integer figures), which makes it possible to use these categories as a quantitative variable in regression analyses. Parity, birth weight, and the BMI of the subject and the educational level of the mother were studied as possible confounders because they are known to be related, as well as BF duration, to body fat mass in later life [21,22]. During the first visit to the Child Health Care clinics the birth weight of the subject and the age and parity of the mother were recorded. Data on the BMI and educational level of the mother were obtained from the mothers by questionnaires. Educational level was categorized as low, medium or high, which was categorized on the basis of the highest level of the education completed by the mother ('low' is primary school and/or secondary school; 'medium' is vocational education and 'high' is higher vocational education and/or university).

### Outcome variables

The BMI (in kg/m^2^), WC (in cm) and WHR were analyzed as continuous outcome variables. Characteristics of the dietary behaviour were obtained by postal questionnaires, composed on the basis of validated questionnaires [23,24]. They concern the frequency of eating breakfast and meals, the consumption of fruit, vegetables, oil-fried snacks such as French fries and croquettes, energy-rich snacks, and the drinking of sweet beverages and alcohol. Sweet beverages are defined as soda or fruit juice, because both contain a lot of sugars [25]. Energy-rich snacks are defined as the consumption of French fries, cakes or candy bars, all containing at least 200 kilocalories per 100 grams: they are among the foods with the highest percentage of saturated fats [25]. The characteristics of the dietary behaviour were dichotomized in healthy and non-healthy outcomes, according to the criteria of the Dutch Nutrition Center (Table 1).

**Table 1:** Dichotomization of outcome variables of dietary behaviour at young adulthood*.

Outcome variable	Unhealthy outcome	Healthy outcome
Breakfast frequency	<5 times/week	≥5 times/week
Meal frequency	<3 times/day	3 times/day
Consumption of fruit	<7 days/week	7 days/week
Consumption of vegetables	<7 days/week	7 days/week
Consumption of fruit or vegetables	<7 days/week	7 days/week
Consumption of fried snacks	> 1 time/week	≤1 time a week
Consumption of other energy-rich snacks	every day	not every day
Consumption of types of snacks	every day	not every day
Consumption of sweet beverages	every day	not every day
Alcohol consumption	>2 glasses/day	≤2 glasses/day

* based on the criteria of the Dutch Nutrition Center (http://www.voedingscentrum.nl/nl/eten-gezondheid)

### Statistical analyses

Mean and frequency differences in the characteristics of the young adults by gender were examined using *t*-tests and χ^2^tests. Logistic regression analysis was applied to study the relationship between the duration of exclusive BF and several aspects of the dietary behaviour. We used linear regression to predict BMI, WC and WHR at adulthood from BF duration at infancy. Analyses were adjusted for gender and age by including them in the regression model. A variable is considered to be a confounder if by adding the variable the regression coefficient of the independent variable changes by more than 10%. As the studied population was not representative of the original population for gender, for the mother's age at the birth or for BF duration, the interaction of these variables with the other independent variables have been studied as well. Statistically significant interaction terms (if *p *< 0.05) were retained in the final model. In addition, we examined whether adding the quadratic term of the BF duration significantly improved the description of the relationship of BF duration with the outcome variables at a level of *p *= 0.05. If significant, this means that the protective effect of BF duration gradually increases (a positive sign of the regression coefficient) or gradually decreases (a negative sign of the regression coefficient). Finally, the aspects of the diet that were found to have a statistically significant relationship with BF were added to the model that explained BMI, WC and WHR, to assess if these aspects mediated the relationship between BF and body fat or visceral fat, according to the causal steps approach of Baron and Kenny [26]. All the statistical analyses were performed with the help of SPSS 15.0.

## Results

In Table 2 characteristics of the population are shown by gender. The mean age of the young adults was 23.1 years (SD 2.9). WC and WHR at adulthood were significantly different for males and females. Dietary behaviour at adulthood also differed significantly for males and females. For most outcomes females show a healthier dietary pattern, except for the consumption of sweet beverages and the consumption of 'other energy-rich snacks', such as candy bars, cake and French fries.

**Table 2:** Demographic background variables, BF duration and dietary factors by gender (n = 810)

	Males (n = 340)		Females (n = 470)	
***Baseline characteristics***	**N**	**Mean (SD)**	**N**	**Mean (SD)**

Age*	340	23.2 (2.9)	470	23.1 (2.9)
Birth weight**	340	3520.6 (501.6)	470	3360.9 (501.6)
Age of mother at birth*	340	27.2 (4.2)	470	26.7 (4.1)
BMI of mother*	306	25.1 (4.0)	426	25.3 (4.0)
BMI at adulthood*	307	23.0 (3.3)	430	23.5 (3.8)
WC at adulthood**	307	84.3 (9.6)	430	79.1 (10.1)
WHR at adulthood**	307	0.86 (0.08)	430	0.79 (0.06)
	**N**	**%**	**N**	**%**
Educational level of the mother*	289		405	
Low		46.4		53.1
Medium		37.7		30.9
High		15.9		16.0
Firstborn*	340	43.2	470	38.3
Exclusive BF duration in days (months, in rounded figures)*	340		470	
0-14 (0)		48.5		47.9
15-44 (1)		13.2		16.0
45-74 (2)		10.0		8.9
75-104 (3)		3.8		5.1
105-134 (4)		4.7		3.4
135-164 (5)		3.2		3.0
≥ 165 (6)		16.5		15.7
***Dietary factors at adulthood***	**N**	**%**	**N**	**%**
Having breakfast ≥ 5 times a week**	337	73.0	468	85.3
Having meals 3 times a day**	335	44.2	464	56.9
Consumption of fruit 7 days a week**	334	26.9	469	36.7
Consumption of vegetables 7 days a week*	336	52.7	468	58.8
Consumption of sweet beverages, not every day**	332	74.1	464	59.9
Consumption of snacks such as French fries and croquettes less than once a week**	340	63.8	470	84.7
Other energy-rich snacks, such as candy bars, cake and French fries, not every day**	335	16.7	468	8.5
Alcohol ≤ 2 consumptions a day**	310	42.6	394	59.4

Differences between males and females by *t*-tests and χ^2^-tests: *non-significant,**p < 0.05

In Table 3 the relationship between BF duration in months and dietary outcomes at young adulthood are shown. A significant dose-response relationship for exclusive BF was found with breakfast frequency and the consumption of snacks such as French fries and croquettes, also after correction for age, gender and possible confounders, with an OR of respectively 1.16 (95% CI 1.06-1.27), and 1.20 (95% CI 1.12-1.30). In none of the investigated relationships did gender or age appear to be effect modifiers. Adding the quadratic function of the BF duration did not improve the models (*p *> 0.05).

**Table 3:** Odds ratios (OR) of exclusive BF duration (in months) for favorable dietary outcomes at young adulthood (n = 810)

Favorable dietary outcomes at young adulthood for:	Crude OR	95%CI	Adjusted OR for age and gender	95%CI	Adjusted OR for confounders	95%CI
Having breakfast	**1.16**	**1.06-1.26**	**1.16**	**1.06-1.27**	**1.16 --**	**1.06-1.27**
Having 21 meals a week	1.03	0.96-1.09	1.01	0.95-1.08	1.03^A^	0.96-1.11
Consumption of fruit	1.05	0.98-1.12	1.05	0.98-1.13	1.02^B^	0.95-1.10
Consumption of vegetables	**1.08**	**1.02-1.15**	1.07	1.00-1.14	1.05^A^	0.98-1.13
Consumption of sweet beverages	1.00	0.93-1.06	1.02	0.96-1.10	0.99^B^	0.91-1.06
Consumption of snacks such as French fries and croquettes	**1.12**	**1.05-1.19**	**1.20**	**1.12-1.30**	**1.15**^A^	**1.06-1.25**
Energy-rich snacks, such as candy bars, French fries and cake	1.02	0.93-1.12	1.06	0.96-1.17	1.01^B^	0.90 - 1.13
Consumption of alcohol	1.02	0.96-1.09	1.05	0.98-1.14	1.07^A^	0.99-1.15

Significant relationships (p < 0.05) are printed in bold

Effect modification by gender, BF duration and age of the mother were not significant in any of the investigated relationships

-- no additional confounders found, ^A^adjusted for educational level mother, ^B^adjusted for BMI mother and educational level of mother

In Table 4 the relationship between the BF duration in months and BMI, WC and WHR are shown. In addition, the effects of adding the variables breakfast frequency and snack consumption on the regression coefficients of the BF duration are shown. As breakfast frequency and snack consumption had no significant relationship with BMI, WC and WHR (*p *> 0.05), and these variables did not significantly influence the regression coefficients of BF duration, no mediation could be shown for breakfast frequency or snack consumption. As these variables did not fulfill the third condition to be defined as mediator[26], the fourth step of the causal steps approach was not performed.

**Table 4:** Relationship between exclusive BF duration (in months) and a. BMI, b. WC, c. WHR at young adulthood in linear regression analyses (n = 710)

		BMI			WC			WHR		
**Model**	**n**	**β (SE)**	***P***	***Adj. *R**^**2**^	**β (SE)**	***P***	***Adj. *R**^**2**^	**β (SE)**	***P***	***Adj. *R**^**2**^

1. Univariate	710	-0.19 (0.01)	<0.001	0.01	-0.59 (0.17)	0.001	0.02	-0.004 (0.001)	0.006	0.01
2. Model 1 adjusted for gender and age	710	-0.19 (0.06)	0.002	0.02	-0.58 (0.17)	<0.001	0.07	-0.003 (0.001)	0.003	0.17
3. Model 2 adjusted for confounders*	605	-0.13 (0.06)	0.042	0.08	-0.39 (0.18)	<0.001	0.12	-0.003 (0.001)	<0.001	0.18
4. Model 3 and adjustment for breakfast	601	-0.14 (0.06)	0.033	0.08**	-0.42 (0.18)	0.020	0.12**	-0.003 (0.001)	0.043	0.18**
5. Model 3 and adjustment for snack consumption	605	-0.13 (0.06)	0.044	0.08**	-0.38 (0.18)	0.031	0.13**	-0.002 (0.001)	0.050	0.18**
6. Model 3 and adjustment for breakfast and snack consumption	601	-0.14 (0.06)	0.034	0.08**	-0.41 (0.18)	0.021	0.13**	-0.002 (0.001)	0.054	0.18**

*The models were adjusted for birth weight, BMI of the mother and educational level of the mother; interactions had no significant relationship with the outcomes.

**No significant changes in explained variance in comparison to model 3, so no effect modification was shown.

## Discussion

Our results show a negative dose-response relationship for the exclusive BF duration with all three outcomes, BMI, WC and WHR. This consistently confirms the hypothesis that the duration of exclusive BF is protective against fat mass in later life. For every month of exclusive BF the BMI, WC and WHR decreased by respectively 0.14 kg/m2, 0.42 cm and 0.003. This implies that for young adults who have been breastfed for 6 months or longer, the BMI, WC and WHR are on average respectively 0.84 kg/m^2^, 2.52 cm and 0.018 lower than for those who have not been breastfed at all. Our findings confirm the results of several studies that assessed the effect of BF on BMI at a later age [9,10,27-31]. The protective effect of BF against overweight may be small, but it is remarkable that the relationship with BF still exists at adulthood, and even protects against visceral obesity. Moreover, at a population level even small effects of BF on fat mass or visceral fat mass may still be relevant for the prevalences of comorbidity of overweight or obesity. We have also shown that the duration of exclusive BF has a dose-response relationship with breakfast frequency and snack consumption at adulthood. Although the relationships between BF duration and the other dietary factors were not statistically significant, all results point to a positive relationship between BF duration and healthy dietary outcomes. However, we could not show that breakfast frequency or snack consumption mediated the relationship between BF duration and fat mass. Remarkably, a significant difference has been found for males and females for several dietary factors. This possibly refers to gender peer effects, which has also been found by others [32].

Only a few studies have been carried out on the relationship between BF and dietary behaviour at later ages or on the mediating effects of dietary behaviour on the relationships of BF with anthropometric variables [15,19,33-35]. As far as we know, only one other cohort study reported on the relationship between BF and dietary behaviour for young adults [35]. In this cohort study, no relationship was shown between BF duration and dietary behaviour at 18 years of age and no mediating effect of dietary behaviour was shown on the relationship between BF duration and measures of overweight. Notwithstanding the high number of included subjects in this cohort, the effects of BF might have been masked by the frequent early introduction of teas, herbal drinks and fruit juices, impeding the study into the effects of exclusive BF [35].

Although most relationships between BF duration and healthy dietary outcomes at adulthood in our study were not statistically significant, they all point to a positive relationship between BF duration and healthy dietary outcomes. This is in line with studies that revealed relationships between BF and several healthy dietary outcomes at later ages: it has been shown that BF is positively related to fruit and vegetable intake at 2-6 years and at 8 years [15,16], to fruit intake by 6-14 year olds [34], and negatively related to the intake of sweetened drinks and added sugars at 12 months of age [19], and intake of white bread, carbonated soft drinks, chocolate bars and fried snacks at 8 years of age [16]. As we studied the relationships until adulthood, it might be that other exposures during later phases in life masked these associations. There are several explanations for the relationships between BF (duration) and healthy dietary outcomes. They might be due to a confounding effect of a higher health consciousness in mothers that breastfeed their child, implying that these mothers will offer a more healthy diet to their children in general[36]. Another explanation may be that food preferences may partly be induced by the variety of flavors offered by breast milk, and the resulting easier acceptance of fruit and vegetable flavors and new solid food by breastfed children in later life, while children that received formula milk accept non-sweet flavors less easily [14,37,38].

Finally, our study results are also in line with another Dutch study that also did not show mediation by dietary factors on the BMI at the age of 7 [16]. The reasons for these similar findings might be that a mediating effect of dietary factors does not exist, that the effects of the mediators are so small that very large study samples are needed to show them, or that the dietary factors studied were too much of a simplification of the dietary behaviour as a whole.

One of the strengths of our study is that the data were prospectively collected from birth onwards, which excludes the possibility of recall bias. Another strength was that we could focus entirely on the exclusive BF duration, implying that the duration of mixed feeding (BF and bottle feeding) could not affect the results. In addition our data enabled us to study proxies of visceral fat, which also appeared to have a dose-response relationship with BF duration. As visceral fat mass is associated with metabolic syndrome and type 2 diabetes [39], a longer exclusive BF duration could be one of the first steps in the primary prevention of these comorbidities.

A limitation of our study was that the value of the collected data on food frequency is limited as they possibly only partly reflect caloric and nutrient intake and the total consumption of fatty food, both of which are highly correlated with fat mass. Therefore we recommend initiating research in which the feeding pattern is studied on the basis of a validated food questionnaire, such as the Food Frequency Questionnaire [40]. Also we had to deal, as a consequence of loss to follow-up, with the fact that the subjects included in this study were not representative of the original cohort with regard to gender, the duration of exclusive BF and the age of the mothers. However, this probably will not have influenced the results as no effect modification has been shown for these variables.

## Conclusion

Our study results indicate that a significant protective dose-response effect of the exclusive BF duration exists on outcomes that approximate total body fat mass as well as visceral fat mass. Although the protective effect is small, on a population level the impact on prevalences of type 2 diabetes and cardiovascular diseases may still be relevant. Because mediation of diet factors were not shown, it should not be precluded that the beneficial effects of BF on later body composition and dietary behaviour evolve by different pathways. Our study results underline the health recommendations by the WHO [41] to exclusively breastfeed children for a duration of at least 6 months.

## Competing interests

The authors declare that they have no competing interests.

## Authors' contributions

MLADK has made substantial contributions to conception and design, acquisition of data as well as to interpretation of data to analysis and drafted the manuscript. RAH and CMR have made substantial contributions to conception and design, and revised the manuscript critically for important intellectual content. JPVW and SVB revised the manuscript critically for important intellectual content. MPJB have contributed to analysis and interpretation of data, and has been involved in revising manuscript critically for important intellectual content. All authors read and approved the final manuscript.

## Pre-publication history

The pre-publication history for this paper can be accessed here:

http://www.biomedcentral.com/1471-2431/11/33/prepub
